# Selecting Appropriate Reference Genes for Quantitative Real-Time Polymerase Chain Reaction Studies in Isolated and Cultured Ocular Surface Epithelia

**DOI:** 10.1038/s41598-019-56054-1

**Published:** 2019-12-23

**Authors:** Sara I. Van Acker, Zoë P. Van Acker, Michel Haagdorens, Isabel Pintelon, Carina Koppen, Nadia Zakaria

**Affiliations:** 1 Antwerp Research Group for Ocular Science, University of Antwerp, Wilrijk, ANT Belgium; 2 Laboratory of Membrane Trafficking, VIB-KU Leuven Centre for Brain & Disease Research, Leuven, VBR Belgium; 3 Department of Ophthalmology, Antwerp University Hospital, Edegem, ANT Belgium; 4 Laboratory of Cell Biology and Histology, University of Antwerp, Wilrijk, ANT Belgium

**Keywords:** Adult stem cells, Reverse transcription polymerase chain reaction

## Abstract

The introduction of tissue engineering has allowed scientists to push the boundaries and treat seriously damaged ocular surface epithelia. They have managed to do this through the development of biological substitutes that restore, maintain or improve tissue function. To ensure the generation of a therapeutically safe and effective graft, knowledge on the transcriptional profile of native and cultured ocular surface epithelia is of undeniable value. Gene expression studies are, however, only as reliable as their proper selection of internal reaction controls or reference genes. In this study, we determined the expression stability of a number of reference genes: *18s rRNA*, *ACTB*, *ATP5B*, *CyC1*, *EIF4A2*, *GAPDH*, *RPL13A*, *SDHA*, *TOP1*, *UBC*, and *YWHAZ* in primary isolates as well as in *ex vivo* cultured ocular surface epithelia explants (day 0 and/or day 14). Expression stability of the reference genes was assessed with both the geNorm and NormFinder software that use a pairwise comparison and a model-based approach, respectively. Our results extend the general recommendation of using multiple reference genes for normalization purposes to our model systems and provide an overview of several references genes that are likely to be stable in similar culture protocols.

## Introduction

**Figure 1:**
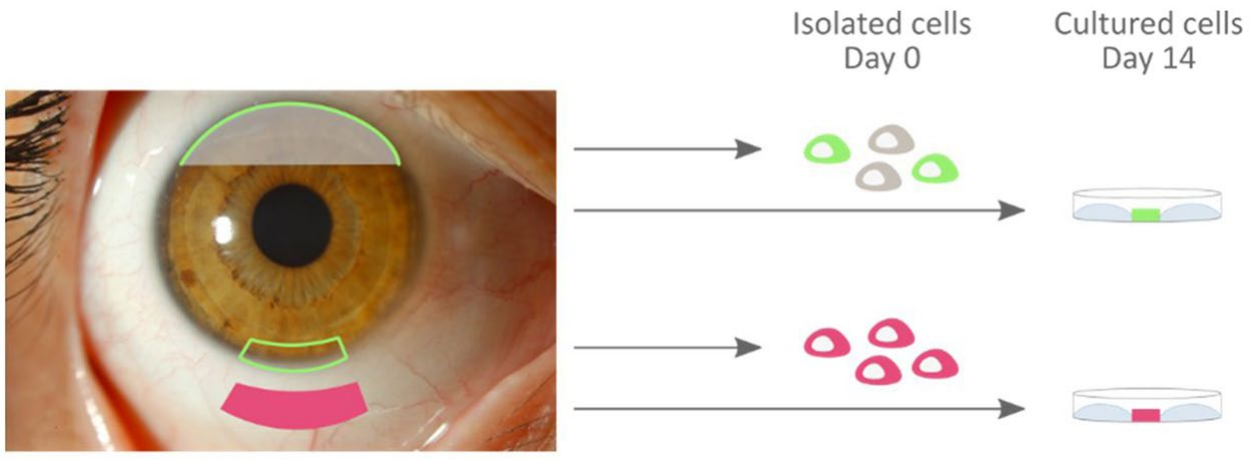
Schematic representation of the experimental culture set-up to obtain mRNA from isolated (day 0) and cultured (day 14) ocular surface epithelia. The steps to isolate human cadaveric donor tissue are in chronological order: the dissection of the inferior and superior bulbar conjunctival region (pink form, inferior region) and the removal of the ocular globe. After the isolation of inferior and superior keratolimbal biopsies from the ocular globe (green framework, inferior region), the corneolimbal epithelium can be trephined (green line = limbus, grey transparent surface = cornea). To determine the mRNA profile of *in vivo* limbal- (green), corneal- (grey), and conjunctival (pink) cells, the extracellular content of corneolimbal epithelium and conjunctival biopsies is enzymatically digested. The resulting single cell suspension is then lysed to allow mRNA collection. In parallel, limbal- and conjunctival explant cultures are initiated from keratolimbal and conjunctival biopsies. After a culture period of 14 days, confluent cultures undergo cell lysis to release their mRNA content. Ocular surface photograph © 2019 Zoë Dupon.

The World Health Organization recently published its first report on vision^1^, the most dominant of our senses when it comes to the perception and interaction of our daily life. The report emphasizes on the substantial economic burden that is caused by damage to the anterior part of the eye or ocular surface, not forgetting the social implications for both the patient and his/her surroundings. Unfortunately, damage to the eye surface still makes out a substantial share of all the causes of blindness^1^. The ocular surface is anatomically composed of the cornea, surrounding conjunctiva, and overlying tear film (Fig. 1)^2^. Homeostasis and renewal in the ocular surface epithelia is sustained through corneal and conjunctival stem cells^3,4^. The corneal stem cell compartment is concentrated in a histologically distinctive ring-like junction between the cornea and conjunctiva, designated as limbus (Fig. 1)^5^. On the contrary, conjunctival stem cells are located throughout the conjunctival basal layer with a higher proportion in the greater physical protected regions^3^. In severe ocular surface disorders such as chemical or thermal burns, Stevens-Johnson syndrome, and ocular cicatricial pemphigoid, the regenerating capacity of the ocular surface becomes exhausted as the stem cell content decreases^6^. The resulting scar formation and epithelial cell loss leads to the development of a hostile environment, which can be experienced by patients through symptoms of discomfort, pain, blurred vision, and even blindness^6,7^. Through the introduction of tissue engineering, the prospect to restore a healthy ocular surface in patients with severe ocular surface disorders has become a reality. Corneal epithelium regeneration and visual recovery have indeed been established through the transplantation of cultured limbal sheets derived from a very small stem cell biopsy as of 1997^8^. However, as the ocular surface works as a functional unit^9^, it is not surprising that the success rate of corneal regeneration is found to be positively correlated with the presence of a healthy conjunctiva and tear film^10,11^. Therefore, it is essential to first normalize the tear film and rehabilitate the eyelid and fornix in patients with combined corneal and conjunctival pathology, using *ex vivo* cultured conjunctival tissue grafts, before proceeding with corneal reconstruction^10,11^.

To assure regeneration and functional re-instatement of the cornea and conjunctiva, one aspect that needs to be controlled is the gene expression profile of the cultured limbal- and conjunctiva-derived cells. This profile should be identical to or resembling the profile of *in vivo* ocular surface epithelia to the utmost extent possible. The transcript expression levels of relevant genes can be measured simultaneously with quantitative reverse transcription PCR (RT-qPCR)^12^. As various sources of variability exist throughout the RT-qPCR protocol, data should be normalized to allow for an accurate comparison of expression levels between different samples and conditions. A widely used strategy is to normalize mRNA levels to the ones of stable reference gene(s)^13^.

Housekeeping genes are generally used as a reference, considering these genes are required for the maintenance of basal cell functions that are essential for every cell type across conditions^14^. Hence, due to their cellular indispensability, a stable expression is expected regardless the cell’s differentiation type, cell cycle stage, developmental stage or tissue environment^14^. The ultimate reference gene - characterized with an invariant expression in all cells and across different physiological and experimental conditions - has, however, not yet been identified. Therefore, selecting a set of housekeeping genes that as a whole provides a stable reference, is of utmost importance to obtain representative results and to detect differences in expression profiles between conditions. False positive or negative errors can for instance occur when the expression of reference gene(s) randomly fluctuates between samples or when the experimental set-up induces a directional expression change^15,16^. Furthermore, normalization against a single reference gene can lead to erroneous expression differences of more than 3-fold the actual value in 25% of data normalizations^17^. Hence, as stated in the Minimum Information for publication of Quantitative real-time PCR Experiments (MIQE) guidelines, this normalization strategy is unacceptable unless adequate justification is provided^18^. Despite the MIQE guidelines, recent studies continue to use such an unvalidated single gene normalization strategy far too often.

The selection of an appropriate set of reference genes is thus depending on the experimental set-up. Even a small change in culture conditions can alter the optimal set of stable housekeeping genes. An illustration hereof is the observed difference in expression levels of commonly used reference genes (such as 18s ribosomal RNA (*18s rRNA*), glyceraldehyde 3-phosphate dehydrogenase (*GAPDH*), and β-actin (*ACTB*)) when the epidermal growth factor is added to the culture medium of placenta-derived stem cells^19^. Hence, molecular studies investigating the stability of reference genes cannot dictate which genes should be implemented when experimental set-ups do not align. However, the top-ranked genes of studies closely aligning with the own protocol are of valuable information to deduce a list from that can be verified experimentally.

To date, preferred reference genes for expression normalization purposes of ocular surface epithelia have solely been identified for tissue samples obtained from frozen tissue sections through laser-assisted microdissection^20^. The following stable reference gene pairs have been determined to provide in a stable reference within the corresponding regions; hypoxanthine guanine phosphoribosyl transferase (*HPRT1*) - TATA-box binding protein (*TBP*) genes (cornea), β-glucuronidase (*GUSB*) - peptidylprolyl isomerase (*PPIA*) (limbus), β2-microglobulin (*B2M*) - *PPIA* (limbal epithelial crypts), and ribosomal protein large P0 (*RPLP0*) - phosphoglycerate kinase (*PGK1*) (conjunctiva). When all the regions are combined, the *PPIA*-*RPLP0* pair shows the lowest expression variability^20^. However, no validation reports have yet been published on the accurate normalization potential of reference genes in cultured ocular surface epithelia.

The aim of this study is to identify the most stable reference genes to normalize expression levels in six different conditions, distinguishable by cell type and time point (Fig. 1). Human samples of two ocular surface epithelia types (i.e. limbus and conjunctiva) are included, encompassing isolated cells (day 0), cultured cells (day 14) or both sample types combined (day 0–14). We validated the expression stability of 12 commonly used reference genes, i.e. *18s rRNA*, *ACTB*, ATP synthase F1 complex β-subunit (*ATP5B*), *B2M*, cytochrome C1 (*CyC1*), eukaryotic translation initiation factor 4A2 (*EIF4A2*), *GAPDH*, *RPL13A*, succinate dehydrogenase complex subunit A (*SDHA*), DNA topoisomerase I (*TOP1*), ubiquitin C (*UBC*), and tyrosine 3-monooxygenase/tryptophan 5-monooxygenase activation protein ζ polypeptide (*YWHAZ*). The corresponding expression stability of the reference genes is assessed using the geNorm and Normfinder software. To the best of our knowledge, we are the first to determine the most suited reference genes for normalization of expression levels in limbal- and conjunctival cultures, whether or not combined with isolated primary samples.

## Results

### Expression profiles of candidate reference genes

We assessed the expression levels of 12 reference genes (quantification cycle (Cq) values) in samples of primary limbal and conjunctival cells at two time points: day 0 and after 14 days in culture (Table 1). The majority of the reference genes had a Cq value between 15 and 25. The lowest Cq value reported belonged to *18s rRNA* with a value below 10 in each group. Values higher than 25 are noted in some conditions for *CyC1*, *SDHA*, and *TOP*1. Considering the standard deviation (SD) as a measure of variation, we obtained a first indication of the expression stability of the reference genes. As such, the three most stably expressed genes within the six groups are; (I) limbus day 0: *GAPDH*, *B2M*, and *YWHAZ*, (II) limbus day 14: *EIFYA2*, *CyC1*, and *YWHAZ*, (III) limbus day 0–14: *UBC*/*EIFYA2*, *18s rRNA*, and *GAPDH*, (IV) conjunctiva day 0: *TOP1*, *YWHAZ*, and *ATP5B*, (V) conjunctiva day 14: *YWHA*Z, *B2M*, and *EIFYA2*, and (VI) conjunctiva day 0–14: *TOP1*, *ATP5B*, and *CyC1*. *YWHAZ* is ranked in each time point except in the combined condition (day 0–14), and has therefore a potential stable expression in (cultured) ocular surface epithelia. Of note, the SD values taken across all samples range between 0.23 and 1.52.

**Table 1:** Cq values of 12 candidate reference genes obtained by quantitative reverse transcription PCR in samples of dissociated (day 0) and/or cultured (day 14) cells of limbal or conjunctival origin.

Gene symbol	Limbus			Conjunctiva		
	Day 0	Day 14	Day 0–14	Day 0	Day 14	Day 0–14
*18s rRNA*	9.67 ± 0.64	9.58 ± 0.57	9.63 ± 0.59	8.38 ± 1.11	9.62 ± 0.99	9.00 ± 1.21
*ACTB*	20.02 ± 0.69	18.38 ± 0.38	19.20 ± 1.00	19.36 ± 0.94	17.89 ± 0.93	18.62 ± 1.18
*ATP5B*	23.35 ± 0.76	22.53 ± 0.42	22.94 ± 0.73	23.28 ± 0.81	23.63 ± 0.66	23.45 ± 0.74
*B2M*	22.33 ± 0.45	21.23 ± 0.65	21.78 ± 0.79	19.86 ± 0.96	22.43 ± 0.55	21.14 ± 1.52
*CyC1*	25.29 ± 0.55	23.85 ± 0.29	24.57 ± 0.86	24.73 ± 0.89	24.73 ± 0.74	24.73 ± 0.80
*EIF4A2*	23.21 ± 0.74	23.19 ± 0.23	23.20 ± 0.54	22.71 ± 0.85	23.43 ± 0.61	23.07 ± 0.81
*GAPDH*	20.09 ± 0.44	19.15 ± 0.46	19.62 ± 0.65	19.99 ± 0.89	19.07 ± 0.84	19.53 ± 0.97
*RPL13A*	23.51 ± 0.95	22.15 ± 0.52	22.83 ± 1.02	21.85 ± 0.98	21.48 ± 0.67	21.67 ± 0.84
*SDHA*	25.37 ± 1.00	25.44 ± 0.48	25.40 ± 0.76	25.16 ± 1.21	26.84 ± 0.86	26.00 ± 1.34
*TOP1*	25.48 ± 1.47	23.75 ± 0.35	24.61 ± 1.37	24.01 ± 0.74	24.00 ± 0.62	24.01 ± 0.67
*UBC*	19.79 ± 0.55	19.47 ± 0.51	19.63 ± 0.54	19.17 ± 1.01	20.94 ± 1.20	20.05 ± 1.41
*YWHAZ*	22.31 ± 0.48	21.19 ± 0.31	21.75 ± 0.70	22.49 ± 0.80	20.94 ± 0.38	21.72 ± 1.00

Values are presented as mean ± standard deviation.

### Expression stability analysis

In a next step, we used the geNorm and NormFinder algorithm software to analyse the stability of the proposed reference genes. The lists with the best-ranked genes are summarized in Table 2. Though the same dataset was used as an input, different rankings are obtained with the different software, sharing a small overlap in their top 3. Only three genes were attributed with a high stability across the different methods used: *YWHAZ* (limbus day 0; conjunctiva day 14), *EIF4A2* (limbus day 14), and *ATP5B* (conjunctiva day 0–14). Additional stable reference genes were shared between the two software or one of the two software and a small SD value. Both the geNorm and NormFinder software top-ranked *UBC* (limbus day 0), *TOP1* (limbus day 14), *CyC1* and *ACTB* (limbus day 0–14). Furthermore, of the genes identified by NormFinder as stable, two genes - *YWHAZ* (limbus day 14) and *ATP5B* (conjunctiva day 0) - have a low SD value as well. Both a favourable geNorm ranking and a small SD are obtained for *B2M* (conjunctiva day 14), *TOP1* and *CyC1* (conjunctiva day 0–14). Given the geNorm and NormFinder algorithm to retrieve other stable genes, we will discuss both algorithms separately in the next paragraphs.

**Table 2:** Top ranking of the most stably expressed reference genes in ocular surface epithelia (limbus vs conjunctiva) at different time points (day 0, day 14, and day 0–14).

Top	Day 0					
	Limbus			Conjunctiva		
	SD	geNorm	NormFinder	SD	geNorm	NormFinder
1.	*GAPDH*	*ACTB*	*YWHAZ*	*TOP1*	*RPL13A*	*ATP5B*
2.	*B2M*	*YWHAZ*	*ATP5B*	*YWHAZ*	*CyC1*	*UBC*
3.	*YWHAZ*	*UBC*	*UBC*	*ATP5B*		*EIF4A2*
**Top**	**Day 14**					
	**Limbus**			**Conjunctiva**		
	SD	geNorm	NormFinder	SD	geNorm	NormFinder
1.	*EIF4A2*	*TOP1*	*EIF4A2*	*YWHAZ*	*B2M*	*ATP5B*
2.	*CyC1*	*EIF4A2*	*YWHAZ*	*B2M*	*YWHAZ*	*SDHA*
3.	*YWHAZ*		*TOP1*	*EIF4A2*	*TOP1*	*YWHAZ*
**Top**	**Day 0–14**					
	**Limbus**			**Conjunctiva**		
	SD	geNorm	NormFinder	SD	geNorm	NormFinder
1.	*UBC/EIF4A2*	*YWHAZ*	*CyC1*	*TOP1*	*CYC1*	*ATP5B*
2.	*18s rRNA*	*ACTB*	*ACTB*	*ATP5B*	*ATP5B*	*EIF4A2*
3.	*GAPDH*	*CyC1*	*SDHA*	*CyC1*	*RPL13A*	*UBC*
4.					*TOP1*	

The top three is given for SD and Normfinder, while the optimal number of genes for an accurate normalization – determined by the software itself – is summarized for the geNorm algorithm.

#### GeNorm data analysis

**Figure 2:**
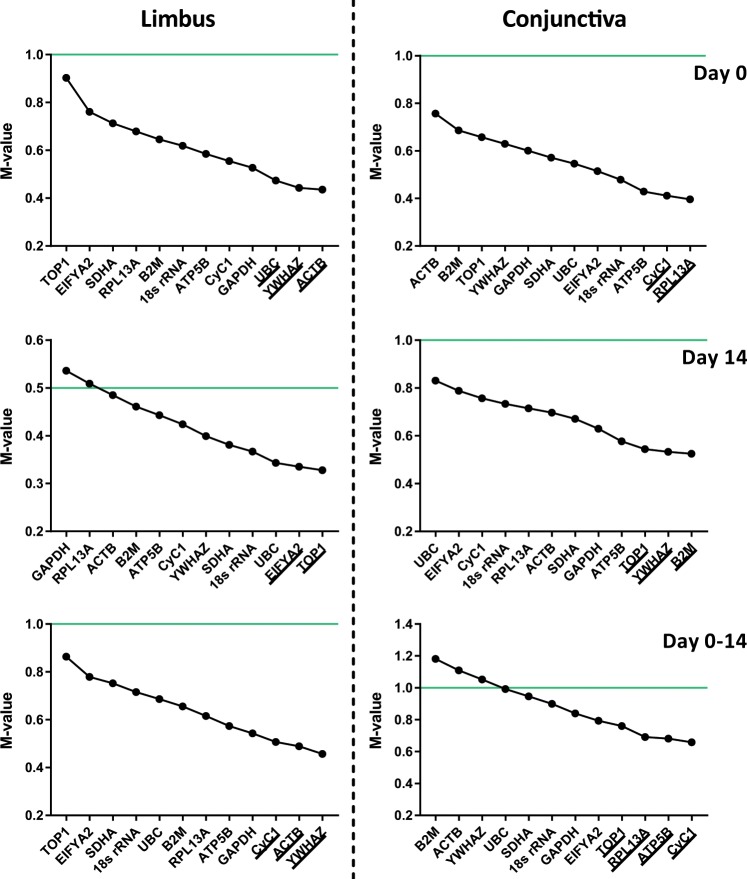
M-value of reference genes, determined by the geNorm software, in isolated (day 0) and/or cultured (day 14) limbal or conjunctival cells. The average expression stability is visualized during stepwise exclusion of the most unstable reference gene, leaving the most stably expressed genes on the right. The specific set of reference genes required to obtain an accurate normalization in each condition is underlined. The green lines represent the threshold of M-values that correspond to suitable reference genes in homogenous samples (M < 0.5) and heterogeneous samples (M < 1.0).

To describe the stability of the reference genes, the geNorm software provides for each gene a corresponding M-value (cfr. Materials and Methods), which are visualized in Fig. 2. The M-value of a suitable reference gene for a homogenous and heterogenous sample should be below 0.5 and 1, respectively^21^. Except for the cultured limbal stem cells, the samples can be characterized as heterogenous. The isolated corneolimbal epithelium contains both limbal stem cells and differentiated corneal cells. Likewise, the isolated and cultured conjunctiva-derived cells consist of conjunctival stem cells, epithelial cells and goblet cells. The corresponding threshold in each condition is depicted in Fig. 2, using green lines. Despite the majority of the M-values falling below the thresholds, we found that *GAPDH* and *RPL13A* lie above the 0.5 upper limit in cultured limbal stem cells. In addition, the M-value of *B2M*, *ACTB*, and *YWHAZ* surpasses the threshold of 1.0 in the isolated and cultured conjunctival cells. Of note, none of the M-values exceeds 1.5, which is defined as the upper limit for candidate reference genes. Information regarding the stability differences can also be obtained from the course of the consecutive M-values. A relative steep initial decline in M-values can be observed after the exclusion of *TOP1* (limbus day 0; limbus day 0–14) and *ACTB* (conjunctiva day 0), reflecting an aberrant expression pattern. Besides the M-value, geNorm also determines the optimal set of housekeeping genes to be used for reliable normalization. This set is underlined and summarized in Fig. 2 and Table 2, respectively. The reference genes *YWHAZ*, *TOP1*, and *CyC1* are shared between limbal- and conjunctiva-derived cells at one or more time points. Furthermore, the *YWHAZ* and *ACTB* gene are selected as optimal at 2 time points in limbal cells, while the *CyC1*, *YWHAZ*, and *TOP1* are the equivalent in the conjunctiva-derived cells.

#### NormFinder data analysis

The stability values of the 12 reference genes calculated by NormFinder are ranked in Table 3. All the reference genes in the top three of the conjunctiva-derived cells are listed in the top three of the limbal conditions as well. The three reference genes ranked at day 0 and day 0–14 in conjunctival cultures were the same for both time points, while only one gene (*YWHAZ*) was shared between the limbal conditions at two time points. Focusing on the low stability values, we found that the values of *TOP1* and *ACTB* correspond well with the relative steep decline observed in the geNorm data analysis. Furthermore, *B2M* is found in the top 4 of the least stable reference genes except for the cultured conjunctival cells (day 14). As the NormFinder software is also able to consider intergroup variation, the best combination of two genes is provided when two or more groups were present. When analyzing day 0 and 14 concomitantly, we found that the combination of *CyC1*/*SDHA* (stability value = 0.093) and *GAPDH*/*TOP1* (stability value = 0.104) let to smaller stability values as compared to values of single reference genes for limbal- and conjunctiva-derived cells, respectively.

**Table 3:** Ranking order of stability values provided by NormFinder.

Ranking order	Day 0				Day 14				Day 0–14			
	Limbus		Conjunctiva		Limbus		Conjunctiva		Limbus		Conjunctiva	
	Gene	SV	Gene	SV	Gene	SV	Gene	SV	Gene	SV	Gene	SV
1	*YWHAZ*	0.218	*ATP5B*	0.211	*EIF4A2*	0.140	*ATP5B*	0.269	*CyC1*	0.118	*ATP5B*	0.117
2	*ATP5B*	0.273	*UBC*	0.239	*YWHAZ*	0.167	*SDHA*	0.287	*ACTB*	0.152	*EIF4A2*	0.167
3	*UBC*	0.290	*EIF4A2*	0.257	*TOP1*	0.186	*YWHAZ*	0.304	*SDHA*	0.156	*UBC*	0.187
4	*CyC1*	0.300	*RPL13A*	0.274	*CyC1*	0.206	*B2M*	0.343	*ATP5B*	0.178	*CyC1*	0.203
5	*18s rRNA*	0.318	*YWHAZ*	0.295	*SDHA*	0.222	*ACTB*	0.358	*GAPDH*	0.182	*RPL13A*	0.205
6	*GAPDH*	0.325	*GAPDH*	0.329	*18s rRNA*	0.264	*GAPDH*	0.407	*EIF4A2*	0.197	*YWHAZ*	0.205
7	*ACTB*	0.381	*CyC1*	0.349	*UBC*	0.269	*RPL13A*	0.411	*RPL13A*	0.209	*GAPDH*	0.206
8	*SDHA*	0.451	*TOP1*	0.362	*ATP5B*	0.274	*18s rRNA*	0.418	*YWHAZ*	0.221	*SDHA*	0.209
9	*B2M*	0.469	*B2M*	0.446	*ACTB*	0.311	*TOP1*	0.423	*UBC*	0.239	*B2M*	0.216
10	*RPL13A*	0.506	*18s rRNA*	0.460	*B2M*	0.337	*CyC1*	0.466	*18s rRNA*	0.249	*TOP1*	0.216
11	*EIF4A2*	0.546	*SDHA*	0.478	*RPL13A*	0.353	*EIF4A2*	0.577	*B2M*	0.370	*18s rRNA*	0.223
12	*TOP1*	1.054	*ACTB*	0.705	*GAPDH*	0.402	*UBC*	0.621	*TOP1*	0.678	*ACTB*	0.223

The 12 reference genes are arranged on decreasing stability and grouped based on their origin (limbus, conjunctiva) and time of lysis (after isolation at day 0, after 14 days of culture or combining samples of both time points for analysis).

SV, stability value.

## Discussion

A thorough appreciation of the characteristic transcriptional profile of the ocular surface provides us valuable information to assist in establishing a tissue-engineered graft that can safely re-instate the epithelial functionality upon transplantation. Accurate gene expression studies on ocular surface epithelia can only be performed when appropriate reference genes are included. Several single genes are already reported for normalization purposes as well as studies using combinations thereof. The *18s rRNA*^22–24^, *ACTB*^25–31^, methyltransferase domain-containing protein 2 (*FTSJD2*)^32^, *GAPDH*^33–53^, and *GAPDH*/*ACTB*/*B2M*/*HPRT1*/*RPL13D* gene^54^ have been used with limbal-derived cells, while *18s rRNA*^24^, *ACTB*^31,55–59^, *GAPDH*^45,53,60–68^, *HPRT1*^69^, and *ACTB*/*GAPDH*/*RPL13A*/*HPRT1*^70^ represent the published reference genes used for conjunctiva-derived cells. In general, an overview of the used reference genes indicates that expression levels are predominantly normalized against *GAPDH*, followed by *ACTB* levels. Their use as internal reaction control is surprising, considering the increasing evidence that the expression of these genes can fluctuate under different experimental set-ups^71^. Moreover, issues concerning the use of the less frequent reported *18s rRNA* as a reference gene has been raised as well^17,71^. The *18s rRNA* represents 20% of the total cellular RNA content^69^. Its transcript levels are therefore considered as highly abundant in comparison to other reference or target mRNA transcripts levels^17,20^. Along with potential expression variability, this could interfere with an accurate normalization^71^.

In this study, we determined the expression stability of *18 s rRNA*, *ACTB*, *ATP5B*, *CyC1*, *EIF4A2*, *GAPDH*, *RPL13A*, *SDHA*, *TOP1*, *UBC*, and *YWHAZ* in isolated and cultured ocular surface epithelia. Based on our results, promising stable reference genes are (I) *UBC*, *YWHAZ*, *ACTB*, and *ATP5B* for isolated limbal cells, (II) *EIFYA2*, *TOP1*, and *YWHAZ* for cultured limbal cells, (III) *ACTB*, *CyC1*, *YWHAZ*, and *SDHA* for isolated and cultured limbal cells, (IV) *ATP5B*, *CyC1*, *EIF4A2*, *RPL13A*, and *UBC* for isolated conjunctival cells, (V) *YWHAZ*, *ATP5B*, *B2M*, *SDHA*, and *TOP1* for cultured conjunctival cells, and (VI) *ATP5B*, *CyC1*, *EIF4A2*, *RPL13A*, *TOP1*, and *UBC* for studies combining isolated and cultured conjunctival cell samples. Of note, the underlined references genes are shared between geNorm and NormFinder. As expected from our literature search, *GAPDH* and *18s rRNA* have not been ranked as stable reference genes in our analyses using both the geNorm and NormFinder algorithms. Hence, our results extend the general recommendation of not using either of these genes as a single gene for normalization purposes in limbal- and conjunctiva-derived cells. On the contrary, *ACTB* was stably expressed in our limbal-derived cells at day 0 and day 0–14. A stable expression pattern, however, does not support a single gene normalization. The optimal number of reference genes to establish an accurate normalization is identified by geNorm and ranges from two to four genes (Fig. 2), depending on the cell type and time point. Hence, according to geNorm, single gene normalization will not provide reliable results in ocular surface epithelial isolations or cultures. Another evident observation entails the limited similarity between the top-ranked genes of limbal- and conjunctiva-derived cells. Despite of the ocular surface epithelia being embedded in the same tear film environment and to have been simultaneously arisen from the Pax6^+^ ectodermal cells during development^72^, they represent two different lineages and should be treated as such when selecting reference genes.

Depending on the experimental set-up, the geNorm and NormFinder top-ranked housekeeping gene list is almost identical or completely different. Given the same crude data set was used as an input, we conclude that these discrepancies are in all probability the result of the different analysis approaches; i.e. geNorm applies a pairwise comparison approach, while NormFinder is based on a model-based approach (cfr. Materials and Methods). As elucidated in Materials and Methods, the pairwise comparison approach is more prone to select correlated reference genes. Among the 12 common reference genes, some are involved in the same pathway or cellular process (Table 4). An example hereof are the three mitochondrial respiratory complexes; *SDHA* (complex II), *CyC1* (complex IV), and *ATP5B* (complex V). Together with two additional complexes, these enzymes are responsible for the production of cellular ATP^73^. As the protein subunits are coded in both the mitochondrial and nuclear genome^73^, an orchestrated crosstalk is required to assure a proper functioning of the respiratory chain^74^. The expression of genes involved in the oxidative phosphorylation are indeed found to be co-regulated^74–76^. However, gene transcription of proteins within each complex are also subjected to an individual fine-tuning mechanism^74^. Keeping in mind the overall master regulation, we expected geNorm to select our mitochondrial gene targets in its top-rankings. Surprisingly, it was NormFinder that selected two out of three mitochondrial housekeeping genes (limbus, day 0–14; conjunctiva, day 14), while geNorm only top-ranked such a combination in one condition (conjunctiva, day 0–14). Hence, at least in our experimental condition, geNorm does not favour this specific co-regulation as expected. Of note, the aforementioned three genes were the only genes that are involved in the same cellular process and co-enlisted by geNorm and NormFinder.

**Table 4:** Characteristics of reference genes and their corresponding primer.

Gene name	Gene symbol	Genebank accession	Anchor nucleotide^*^	Function
18S ribosomal RNA	*18S rRNA*	NM_10098	234	Translation (component of ribosomal 40S subunit)
β-actin	*ACTB*	NM_001101	1194	Cell motility, structure, integrity, and intercellular signalling
ATP synthase, F1 complex, β-subunit	*ATP5B*	NM_001686	1200	Mitochondrial respiratory chain (ATP synthesis)
β2-microglobulin	*B2M*	NM_004048	362	Acquired immune system (component of major histocompatibility complex class I heavy chain)
Cytochrome C1	*CyC1*	NM_001916	929	Mitochondrial respiratory chain (electron transport)
Eukaryotic translation initiation factor 4A2	*EIF4A2*	NM_001967	900	Translation (ATP-dependent RNA helicase)
Glyceraldehyde 3-phosphate dehydrogenase	*GAPDH*	NM_002046	1087	Glycolysis and cellular stress response (cell recovery or apoptosis)
Ribosomal protein L13a	*RPL13A*	NM_012423	727	Translation (component of ribosomal 60S subunit) and inflammation (component of GAIT-complex)
Succinate dehydrogenase complex, subunit A, flavoprotein	*SDHA*	NM_004168	1032	Citric acid cycle and mitochondrial respiratory chain
DNA topoisomerase I	*TOP1*	NM_003286	2361	DNA replication and transcription
Ubiquitin C	*UBC*	NM_021009	452	Ubiquitination
Tyrosine 3-monooxygenase/tryptophan 5-monooxygenase activation protein, ζ polypeptide	*YWHAZ*	NM_003406	2585	Mediator of signal transduction

^*^An anchor nucleotide is defined as a nucleotide contained anywhere within the probe sequence according to the MIQE guidelines.

Including the two different time points and the combination thereof allows us to use the top-ranked genes, after appropriate verification, in both research and clinical/diagnostic settings. Focusing on ocular tissue engineering, gene expressions are often investigated after a specific culture protocol (cfr. day 14 as an end of the culture protocol) and/or compared with the *in vivo* situation (day 0–14). In addition to research purposes, RT-qPCR assays are finding their way into the clinic. Examples hereof are the MammaTyper® test (BioNTech Diagnostics GmbH, Mainz, Germany) and the CervicGen assay (Optipharm, Osong, Republic of Korea), which could complement or improve the current standards in cancer diagnostics^77,78^. Based on the success of RT-qPCR in cancer diagnostics, RT-qPCR is also started to be implemented in other fields, including ophthalmology. Potential biomarkers are examined in impression cytology samples of patients with Sjögren’s syndrome^79^, meibomian gland disease^80^, dry eye syndrome^81^, vernal keratoconjunctivitis^64^, and a collection of different ocular surface disorders^82^. However, despite the MIQE guidelines, single gene normalization has still been applied in these studies. Hence, to generate a good experimental basis for future research, we generated a top-ranking of stable genes in ocular surface samples that can be verified in similar research or clinical settings. Of note, in case the most optimal number of reference genes cannot be determined, one should use the three most stable reference genes to establish a more accurate and reliable normalization as compared to the use of one single reference gene^17^. Lastly, a final quality control of the identified reference genes can always be obtained based on the corresponding Cq- and SD values. Previous studies showed that the Cq value of a suitable reference gene should neither fall beneath 15 or exceed 30, while the SD should not surpass 1.0^83,84^.

To summarize, this study confirms the need of using multiple reference genes for normalization purposes. In addition, we provide several potentially stable reference genes for studies on isolated- and cultured ocular surface epithelia.

## Materials and Methods

### Tissue specimen

Human ocular tissue from 22 cadaveric donors was obtained from the Antwerp University Hospital tissue bank as fresh tissue rejects and processed within 48 hours post-mortem. The donor’s age ranged from 43 to 92 years, with an average of 74 years. Before tissue handling, the cadaveric eye globes and isolated conjunctiva were disinfected for 1 minute in 0.5% and 1% povidone-iodine (pharmacy Antwerp University Hospital), respectively, followed by a quadruple washing step in phosphate buffered saline (PBS). The access and use of human biological material, intended for human medical applications or for scientific research purposes follows Article 12 of the Act of December 19, 2009. This act includes that Articles 10 to 14 of the Act of 13 June 1986 on the removal and transplantation of organs (opting-out) are applicable in the context of the removal after death of organs intended for scientific research or on the removal after death of tissues and cells for medical applications or for scientific research. The opting-out system is based on implicit or presumed consent, i.e. every Belgian is a potential donor unless he/she objected against donation during life. We hereby confirm, that all research and methods were performed in accordance with the relevant guidelines and regulations. The study followed the tenets of the Declaration of Helsinki and was approved by the Ethical Committee of the Antwerp University Hospital (approved EC: 11/2/12).

### Primary human limbal epithelial cell cultures

Limbal biopsies were isolated and processed according to the protocol described by Haagdorens *et al*.^85^. In brief, biopsies were taken from the superior and inferior keratolimbal region (Fig. 1) and washed for 30 minutes in CnT-prime medium (CnT-PR, CELLnTEC, Bern, Switzerland) at 4 °C. Explant cultures were initiated at the air-liquid interface, using CnT-PR medium, and cultured for 14 days at 37 °C and 5% CO_2_. From 2 days onward, cultures were submerged and the culture medium was changed every other day. This work flow results in a negligible fibroblast culture contamination, as previously shown^86^. Prior to RNA extraction from the cultured limbal cells at day 14, limbal biopsies were removed using metal tweezers and cultures were rinsed with preheated PBS at 37 °C. Afterwards, cell lysis was performed according to RNeasy Micro kit guidelines (Qiagen, Hilden, Germany).

### Primary human (corneo)limbal cell sheath isolation

After harvesting limbal biopsies from the cadaveric donor eyes, the cornea and limbus were trephined out of the globe. The isolated cornealimbal disc was soaked in a 1 U/mL dispase solution at 4 °C for 14 hours to remove the corneolimbal epithelium from its underlying tissue. The dispase solution is constituted of 10 mL dispase II solution (2 U/mL, Roche, Sigma-Aldrich, Overijse, Belgium), 2 mL D-sorbitol (Sigma-Aldrich), and 8 mL modified-Supplemented Hormonal Epithelial Medium (SHEM). Modified-SHEM, in turn, is made from Ham’s F12 Glutamax (Life Technologies, Merelbeke, Belgium) and supplemented with 5% fetal bovine serum (Life Technologies), 5 µg/mL insulin-transferrin-selenium (Life Technologies), 2 ng/mL epidermal growth factor (Life Technologies), 0.5% dimethyl sulfoxide (Sigma-Aldrich), 0.5 µg/mL hydrocortisone (Sigma-Aldrich), 10 µg/mL gentamicin (Life Technologies), and 1 µg/mL amphotericin B (Life Technologies). After dispase digestion, the corneolimbal epithelium was gently scraped off, using a dissecting microscope, tweezers and a crescent knife. Epithelial sheets of both eyes were pooled and lysed following the RNeasy Micro kit guidelines.

### Primary human conjunctival epithelial cell cultures

Bulbar conjunctiva from the inferior and superior region was isolated before the ocular globe was removed from the cadavers and disinfected as described in ‘tissue specimen’ (Fig. 1). The disinfected tissue was further cut into 2 × 2 mm explants and placed at the liquid-air surface to initiate outgrowth at 37 °C and 5% CO_2_ for 14 days (Fig. 1). The medium was changed thrice a week and consisted of keratinocyte serum-free medium (Life Technologies) supplemented with 50 µg/mL bovine pituitary extract (Life Technologies), 5 ng/mL recombinant human epidermal growth factor (Life Technologies), 10 µg/mL gentamicin (Life Technologies), and 1 µg/mL amphotericin B (Life Technologies). When a visible outgrowth was obtained, primary cultures were submerged as from that point the explants were likely to remain attached. Explants were removed from culture upon the first signs of fibroblast contamination. After the culture period of 14 days, remaining explants were discarded as well and the primary cultures underwent a PBS wash prior to their lysis, using the RNeasy Micro kit recommendations.

### Primary human conjunctival cell suspension

A homogenous conjunctival epithelial cell suspension was established to extract mRNA from isolated conjunctiva-derived cells (day 0). Briefly, disinfected conjunctival tissue was exposed to 1.2 U/mL dispase solution (Sigma-Aldrich) for 2 hours at 37 °C under continuous agitation. The incomplete attached cells were mechanically recovered, using a cell scraper, and the obtained cell suspension was lysed using the RNeasy microkit, according to the manufacturer’s instructions.

### RNA extraction and quantitative reverse transcription PCR

Total RNA was isolated from limbal and conjunctival cell lysates using the RNeasy microkit guidelines. Preceding cDNA conversion, RNA concentration and purity was evaluated through UV spectroscopy on the Nanodrop^TM^ spectrophotometer (Thermo Fisher Scientific). Up to 7.5 µg RNA was utilized as template for first strand cDNA synthesis following the instructions of the iscript advanced cDNA synthesis kit (Bio-rad), containing dNTPs, oligo(DT), random primers, RNase H^+^ Moloney murine leukemia virus reverse transcriptase and RNase inhibitors. The oligo(DT)- and random primer-mediated reverse transcription was performed on a CFX96 Touch™ Real-Time PCR Detection System (Bio-Rad). The obtained cDNA was diluted to a 5 ng/µL concentration and frozen down (-20 °C) until further use. RT-qPCR assays were performed on a CFX96 Touch™ Real-Time PCR Detection System (Bio-Rad) with following settings; an activation step of 30 seconds at 95 °C, 40 amplification cycles of denaturation (95 °C for 5 sec) and annealing/extension (60 °C for 30 sec). A melting curve analysis was performed as well: from 65 °C to 95 °C at 0.5 °C increments for 5 sec. To identify the most stable reference genes, the geNorm 12 gene kit (PrimerDesign, Southampton, United Kingdom) was used. Characteristics of the 12 reference genes and their corresponding primers can be found in Table 4. Each reaction was performed in a final volume of 20 µL, containing 10 µL SsoAdvanced Universal SybrGreen Supermix (Bio-rad), 1 µL primer (300 nM, PrimerDesign), 2 µL diluted cDNA (10 ng), and 7 µL UltraPure™ DNase/RNase-free distilled water (Thermo Fisher Scientific).

### Gene expression stability analysis

The Cq values of the 12 reference genes were expressed as mean ± SD. To determine the expression stability, the RT-qPCR data was processed using the geNorm software, incorporated in the qbase+ software (Biogazelle), and the NormFinder software (MOMA, Department of Molecular Medicine, Aarhus University Hospital, Denmark). In contrast to the qbase+ software, the input data of NormFinder is required to be on a linear scale. Hence, the raw Cq values were transformed into relative quantities using the ΔΔCt-method. As the lowest Cq value of each reference gene was used, the highest relative value equalled one, while all other values are smaller.

The algorithm of the geNorm and NormFinder software uses a different approach to address the stability of the reference genes^17,87^. The geNorm software applies a pairwise comparison approach, which defines the stability of genes based on the similarity degree of the expression profile. The reasoning behind this approach is that the expression ratio of two stable reference genes is identical across the sample set, regardless of the experimental condition^17^. On the other hand, NormFinder uses an ANOVA-based model to calculate the expression stability of each reference gene based on its intra- and intergroup variation^87^. Both software provide a stability value or M-value, which is irreversibly correlated with the expression stability^17,87^. In addition to the M-value, geNorm also determines the number of genes needed for an accurate normalization. GeNorm will first rank the candidate genes up to the single most stable genes, using their corresponding M-values that are calculated based on the expression ratio of a particular gene with the remaining genes upon stepwise exclusion of the most unstable reference gene. Then, the optimal number of reference genes is identified by validating the pairwise variation between two sequential normalization factors, containing an increasing number of genes. The first normalization factor is calculated starting from the three most stable reference genes. The seven consecutive normalization factors are then determined with the stepwise inclusion of the most stable remaining reference genes. When the variation falls below the 0.15 cut-off, the inclusion of an additional gene is not required as it will not anymore improve the stability of the set of reference genes^17^. When interpreting the output data of the NormFinder software, the reference gene with the lowest M-value is the gene with the smallest intra- and intergroup variance. Hence, NormFinder is less affected by the expression of correlated reference genes as systemic differences between subgroups are taken into account. Of note, when only one group is present and therefore no intergroup variation exists, the intragroup variation is the only variance included in the stability value^87^.

## Data Availability

The datasets used to support the findings of this study are available from the corresponding author on reasonable request.
